# A multicenter round robin test of PD-L1 expression assessment in urothelial bladder cancer by immunohistochemistry and RT-qPCR with emphasis on prognosis prediction after radical cystectomy

**DOI:** 10.18632/oncotarget.24531

**Published:** 2018-02-19

**Authors:** Markus Eckstein, Ralph M. Wirtz, Carolin Pfannstil, Sven Wach, Robert Stoehr, Johannes Breyer, Franziska Erlmeier, Cagatay Günes, Katja Nitschke, Wilko Weichert, Wolfgang Otto, Bastian Keck, Sebastian Eidt, Maximilian Burger, Helge Taubert, Bernd Wullich, Christian Bolenz, Arndt Hartmann, Philipp Erben

**Affiliations:** ^1^ Institute of Pathology, University of Erlangen-Nuremberg, Erlangen, Germany; ^2^ STRATIFYER Molecular Pathology GmbH, Cologne, Germany; ^3^ Institute of Pathology at The St. Elisabeth Hospital Köln-Hohenlind, Cologne, Germany; ^4^ Department of Urology, University of Erlangen-Nuremberg, Erlangen, Germany; ^5^ Department of Urology Mannheim, University of Heidelberg, Mannheim, Germany; ^6^ Department of Urology, University of Regensburg, Regensburg, Germany; ^7^ Institute of Pathology, Technical University Munich, Munich, Germany; ^8^ Department of Urology, University of Ulm, Ulm, Germany

**Keywords:** bladder cancer, PD-L1, checkpoint inhibitors, molecular therapy stratification, immunohistochemistry

## Abstract

**Background:**

Immunohistochemical PD-L1 assessment is currently used to identify responders towards checkpoint inhibitors although it is limited by inter-observer effects. Here, we conducted a multi-center round robin test to prove the possibility of assessing the PD-L1 status by gene expression to avoid inter-observer effects.

**Patients and methods:**

Gene expression of PD-L1 was analyzed in a total of 294 samples (14 cases non-muscle invasive and muscle-invasive bladder cancer; MIBC) in seven centers by a RT-qPCR kit and compared with immunohistochemical scoring of three pathologists (DAKO, 22c3). Both assays were compared towards prognosis prediction in a cohort of 88 patients with MIBC.

**Results:**

PD-L1 gene expression revealed very high inter center correlation (centrally extracted RNA: *r* = 0.68–0.98, *p* ≤ 0.0076; locally extracted RNA: *r =* 0.81–0.98, *p* ≤ 0.0014). IHC Inter-observer concordance was moderate to substantial for immune cells (IC), fair for combined IC/ tumor cell (TC) (IC: κ = 0.50–0.61; IC + TC: κ = 0.50), and fair for TC scoring (κ = 0.26–0.35). Gene expression assessment resulted in more positive cases (9/14 cases positive vs. 6/14 cases [IHC]) which could be validated in the independent cohort. Positive mRNA status was associated with significantly better overall and disease-specific survival (5-year OS: 50% vs. 26%, *p =* 0.0042, HR *=* 0.48; 5 year DSS: 65% vs. 40%, *p* = 0.012, HR *=* 0.49). The 1% IHC IC cut-off also revealed significant better OS (5 year OS: 58% vs. 31%, *p =* 0.036, HR *=* 0.62).

**Conclusion:**

Gene expression showed very high inter-center agreement. Gene expression assessment also resulted in more positive cases and revealed better prognosis prediction. PD-L1 mRNA expression seems to be a reproducible and robust tool for PD-L1 assessment.

## INTRODUCTION

Urothelial bladder cancer (UBC) is one of the 10 most common malignancies worldwide [1]. For decades, the only therapy regimen for metastatic UBC was platinum-based chemotherapy which is accompanied with poor overall [2]. Immunotherapy, in particular, antibodies targeting CTLA4, PD-1 or PD-L1 led to partially spectacular treatment success in patients with several malignancies such as melanoma and non-small cell lung cancer (NSCLC) and renal cell carcinoma [3–6]. Response to these therapies is especially convincing in tumor types with high mutational burden probably owing to an increased number of neoantigens [7, 8]. Several clinical studies investigated the effect of PD-1/PD-L1 targeting antibodies in advanced UBC with promising results. Whereas some of them indicated a PD-L1 expression independent responsiveness [9, 10], other found high PD-L1 expression dependent responsiveness [11–13].

Currently, immunohistochemical (IHC) PD-L1 scoring of immune/tumor cell (IC/TC) is applied for therapy stratification of checkpoint inhibitor therapies. As other semi-quantitative IHC assays it is heavily influenced by assessment subjectiveness leading to relevant inter-observer effects. A broadly known example for this diagnostic misery is Her2/neu scoring in breast cancer which is heavily affected by intra- and inter-observer variability [14]. A recently published harmonization study on PD-L1 scoring in NSCLC revealed acceptable agreement for TC staining, but poor agreement for IC scoring why it is questionable if PD-L1 scoring of UBC will reach acceptable inter-observer agreement for IC scoring [15]. Furthermore, this study also revealed that the four commonly utilized assays exhibit quite different staining patterns.

Therefore, we conducted a multicenter round robin test to (I) analyze correlation between a commonly used immunohistochemical (IHC) PD-L1 assay (22c3, Dako) and a reverse transcriptase quantitative polymerase chain reaction kit (RT-qPCR, CheckPointTYPER©, STRATIFYER Molecular Pathology, Cologne, Germany), to (II) investigate the influence of central and local RNA (C/LRNA) extraction as well as of various thermocyclers (III) on reproducibility in 7 laboratories, and to (IV) compare the positive detection rate and prognostic relevance of both assays. To investigate positive detection rate and prognostic relevance an independent cohort of 88 patients with MIBC was investigated.

## RESULTS

### Immunohistochemistry: Inter-observer variability

**Figure 1:**
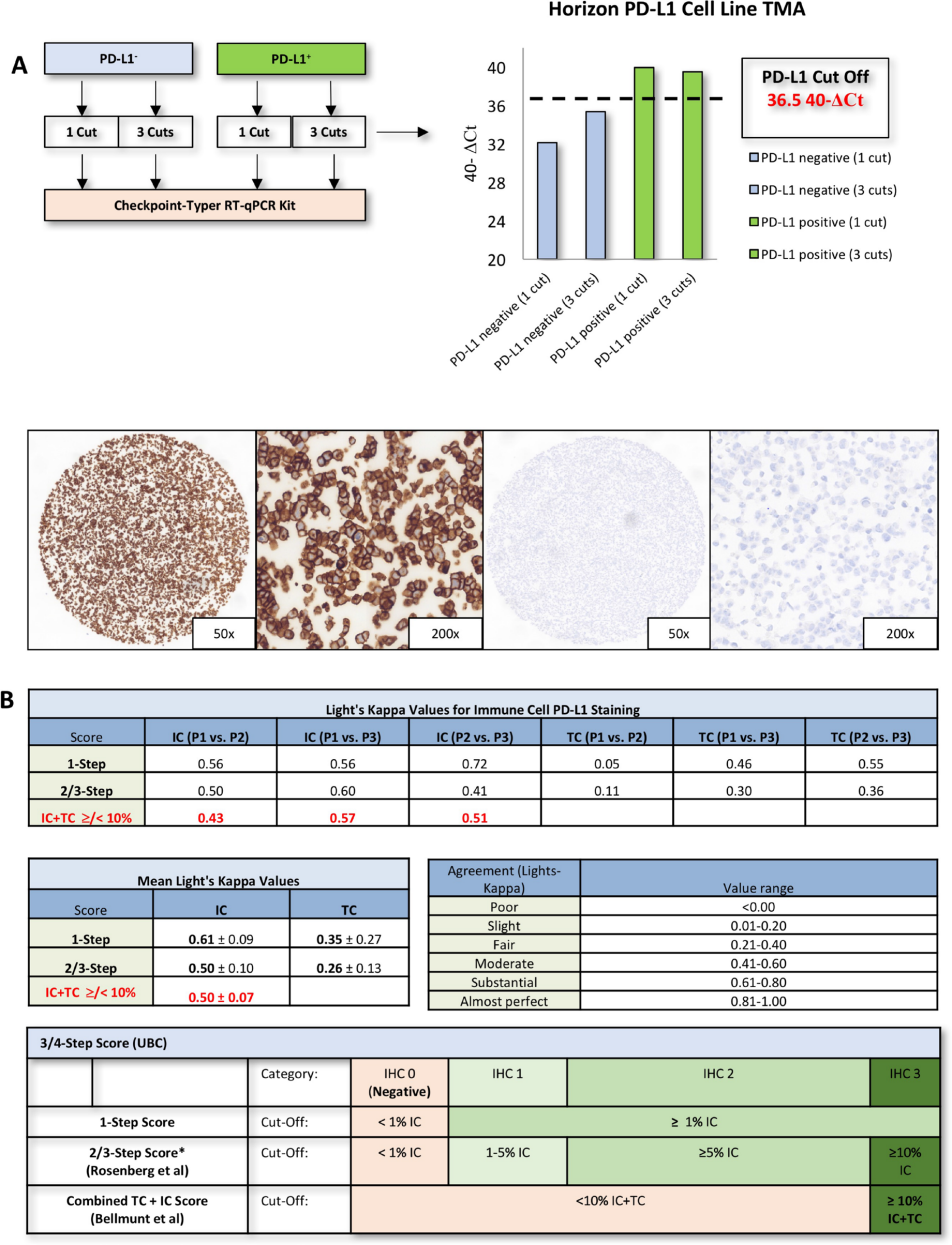
Checkpoint-Typer calibration and inter-observer experiments **(A)** Checkpoint Typer^©^ validating results (analysis of PD-L1 cell lines, Horizon Discovery, Cambridge, United Kingdom). IHC staining of the Horizon positive/negative TMA. PD-L1 expressing cells showed a linear, strong membranous staining while PD-L1 negative cells were completely negative (TMA = tissue microarray; ΔCt = delta “cycle threshold”). **(B)** Light‘s Kappa values for immune cell (IC) and tumor cell staining (TC). All single agreements and the mean agreements are depicted in the table. Overall the IC scoring exhibits a substantial to moderate agreement while TC scoring shows a fair agreement. Combined IC + TC scoring as applied by Bellmunt *et al.* [9], also exhibits moderate agreement. The utilized cut-offs are depicted in the table below. *IHC2 and IHC3 are merged to the IHC2/3 category in IMvigor trial by Rosenberg *et al.* 2016 [12]. Interpretation of Light‘s Kappa values is depicted beside the agreement plots. (P1 = pathologist 1; IHC = immunohistochemistry).

Inter-observer variability for IC staining reached moderate to substantial agreement using 1% cut-off (κ = 0.56–0.72; κ_*mea*_*n =* 0.61 ± 0.09) and moderate agreement using the 2/3-step score (κ = 0.41 to 0.60; κ_*mea*_*n =* 0.50 ± 0.10; Figure 1B) [10, 12]. Inter-observer variability for TC staining revealed slightly worse agreement for both the 1-step score reaching from slight to moderate agreement (κ = 0.05 to 0.55; κ_*mea*_*n =* 0.35 ± 0.27) and the 3-step score reaching from slight to fair agreement (κ = 0.11 to 0,36; κ_*mea*_*n =* 0.26 ± 0.13 Figure 1B). Applying a combined TC + IC cut-off (</≥ 10%) also revealed moderate agreement [9]. Scorings of each single case according to utilized scores (Figure 1B) are depicted in Supplementary Table 1.

### Validation of PD-L1 detection by RT-qPCR

PD-L1^+/-^ cell line derived TMAs (Horizon Discovery) were utilized. Separate measurements of 1 or 3 cuts of negative and positive TMA positions revealed, that the Checkpoint-Typer-Kit is suitable to detect PD-L1 positive and negative FFPE cells reproducibly using a PD-L1 positive threshold of 36.5 40-ΔCt (Figure 1A). Negativity and positivity could be verified by IHC (22c3 assay on horizon positive/negative TMAs; Figure 1A).

### RT-qPCR: Inter-lab variability

**Figure 2:**
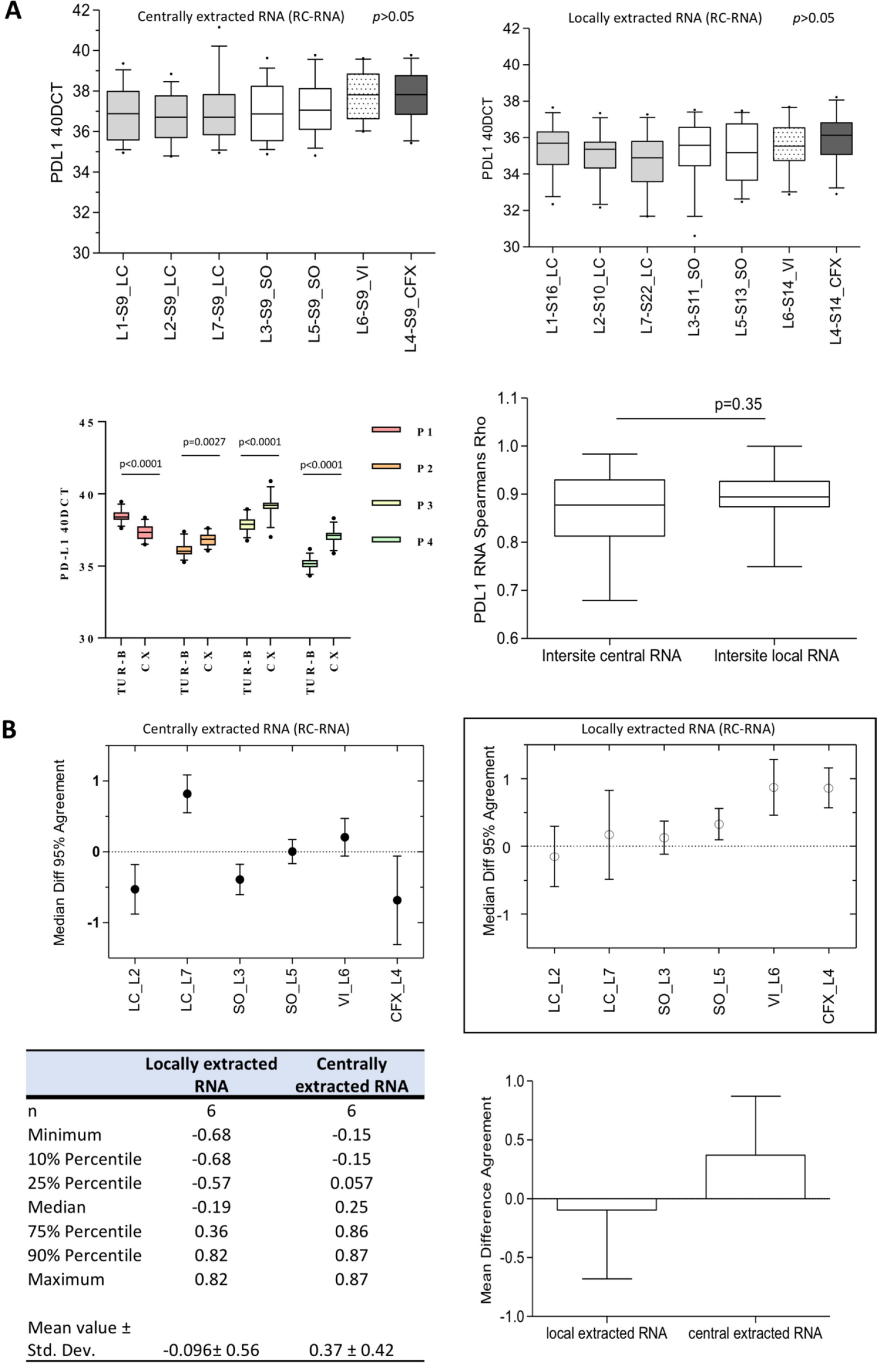
Inter-center reproducibility and agreement of PD-L1 gene expression testing **(A)** There is no statistical significant difference in normalized 40-ΔCt values between the seven participating labs (L1-L7) measuring both CRNA and locally extracted RNA. The thermocycler choice has also no influence. (S9 = section 9; L1 = Lab1; LC = LightCycler; SO = Step-One-Plus; CFX = Biorad CFX; VIA = VIIA7; *p >* 0.05). The lower boxplot indicates that inter-site correlation for CRNA and LRNA is extremely high and did not differ significantly (*p =* 0.35). Explicit pair comparisons of each lab are tabulated in Supplementary Table 2. PD-L1 gene expression of matched pairs of TUR-B and CX differs significantly (P1 and P4 TUR-B > CX; P2, P3 TUR-B < CX). Gene expression between TUR-B specimens and CX specimens differs significantly. **(B)** Summary of all Bland-Altman analysis concerning centrally extracted RNA (1) and locally extracted RNA (2). The mean difference amounts –0.096 ± 0.56 for locally extracted RNA and 0.37 ± 0.42, which indicates an extremely high agreement. (LC = LightCycler; SO = SO = Step-One-Plus; CFX = Biorad CFX; VIA = VIIA7; Std. Dev. = standard deviation). Bland-Altman plots for every lab compared to the reference lab (Lab1) are depicted in Supplementary Table 3.

Every participating lab was able to detect PD-L1 mRNA in the positive control whereas PD-L1 mRNA levels were below the threshold in negative controls. Independent PD-L1 measurements of CRNA (every specimen; section 9) revealed no significantly different normalized 40-ΔCt values between all labs (Figure 2A). The inter-lab correlation for PD-L1 measurements of CRNA was very high in the most pair comparisons (*ρ* = 0.68–0.98, *p <* 0.0076; Supplementary Table 2A). LRNA extractions of the same samples (section 16-22/S16-22) exhibited high correlation with similarly high inter-lab correlations (*ρ* = 0.81–0.97, *p <* 0.0014; Supplementary Table 2B). Usage of different thermocyclers also revealed no significant influence concerning PD-L1 measurements out of CRNA and LRNA (Figure 2A). In contrast to PD-1 mRNA expression, PD-L1 expression was hardly affected by serial sectioning (data not shown).

For further evaluation of inter-lab variability Bland-Altman-analysis was performed setting Lab1 (Kit distributor) as reference. The analysis revealed a strong agreement among all labs (Figure 2B; Supplementary Table 3). No single analysis revealed mean differences of larger/smaller than –0.86 or 0.15 (Figure 2B). In summary, the mean difference amounted –0.096 ± 0.56 (min/max: –0.68 – 0.82) for LRNA and 0.37 ± 0.42 (min/max: –0.15 – 0.87) for CRNA (Figure 2B; Supplementary Table 3).

### Immunohistochemistry and RT-qPCR: Inter-specimen variability

To investigate whether the expression of PD-L1 is diverging between TUR-B and CX specimens we investigated four matched pairs of TUR-B and CX from the same patients. In two matched pairs the gene expression of PD-L1 was significantly higher in the TUR-B than in the CX specimens (*p <* 0.0001; Figure 2A). In the other two pairs it was exactly the opposite (*p =* 0.0027; *p <* 0.0001; Figure 2A). Protein expression detected by immunohistochemistry showed the same relationship with smaller effect size, especially in P1 and P3 (Table 1).

**Table 1:** PD-L1 protein expression in the four matched pairs (P1–P4) of transurethral resection and cystectomy specimens

	TUR-B			CX		
	IC	TC	Combined	IC	TC	Combined
P1	0.5%	0%	0.5%	0%	0%	0%
P2	5%	0%	5%	10%	10%	20%
P3	0%	0%	0%	0.5%	0%	0.5%
P4	30%	0%	30%	5%	0%	5%

Congruent with gene expression, protein expression of PD-L1 is higher in P1 and P2. In P3 and P4 it is exactly the opposite, although the effect size is smaller than in gene expression.

### Concordance of PD-L1 immunohistochemistry and RT-qPCR

**Figure 3:**
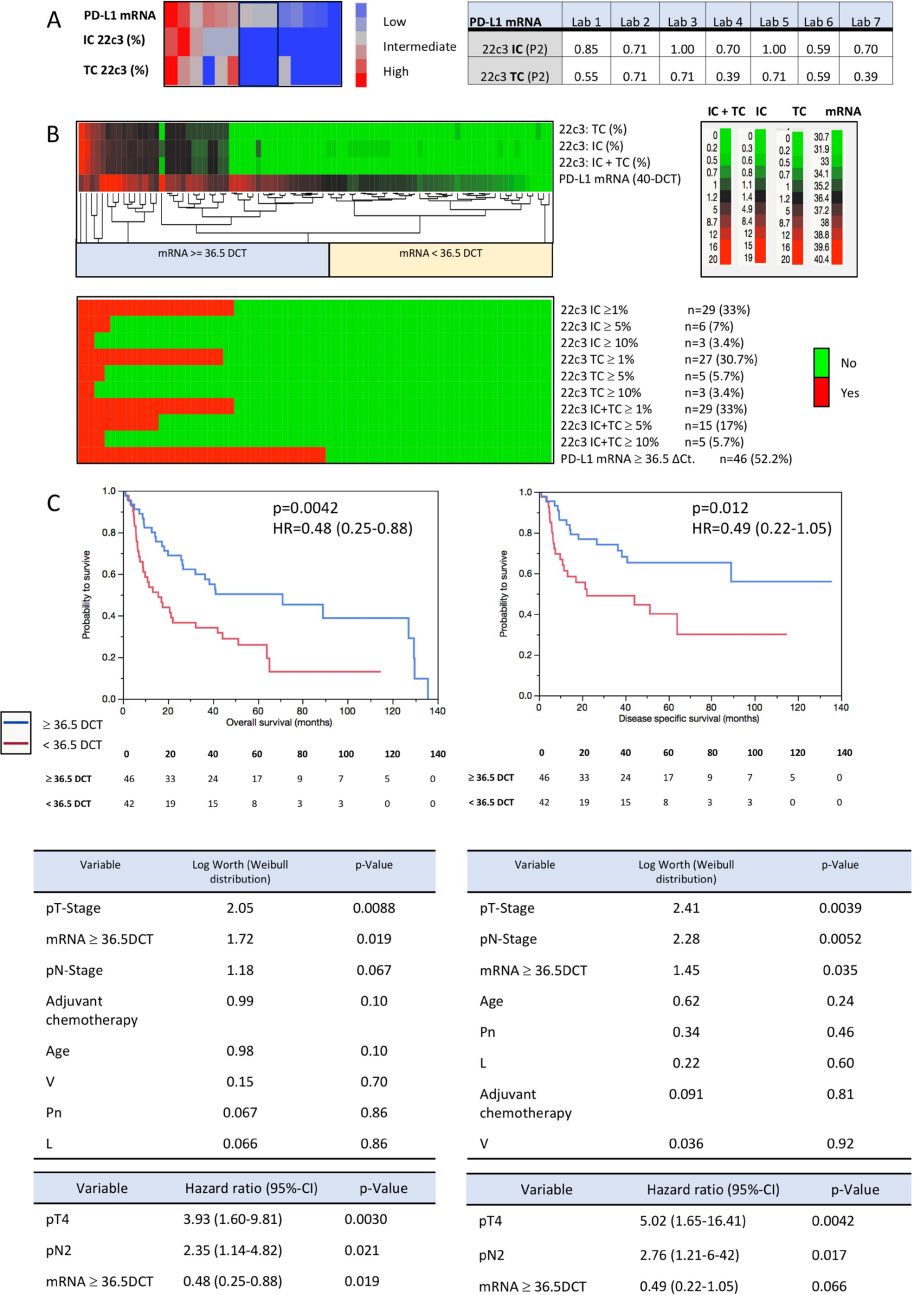
Sensitivity validation and prognosis prediction (PD-L1 gene expression) **(A)** Concordance between PD-L1 mRNA (RC-RNA) and IC/TC staining evaluation of P2. The concordance is presented as Light‘s Kappa values. The concordance is slightly better for IC-staining/mRNA than for TC-staining/mRNA. 2d-hierarchical cluster analysis clustering centrally measured PD-L1 mRNA values against PD-L1 protein expression (continuous percentage values of P2). (IC = immune cells; TC = tumor cells). **(B)** The upper heat map clusters continuous scoring values of immunohistochemistry (IC, TC and TC + IC) and PD-L1 mRNA values (DCT). There is a huge subset of tumors which is classified as mRNA positive (mRNA ≥36.5 DCT; *n =* 17) while they are classified as negative by IHC. The lower heat map demonstrates that this “diagnostic” gap is expanding if other cut-offs are applied (e.g. </ ≥ 10% combined used by Bellmunt *et al*: *n =* 41). **(C)** Kaplan–Meier-analysis revealed that PD-L1 mRNA expression above the cut-off of 36.5 DCT is highly prognostic concerning both, OS and DSS (OS: log rank *p =* 0.0042; HR *=* 0.48 [95%-CI 0.25–0.88]; DSS: log rank *p =* 0.012; HR *=* 0.49 [0.22–1.05]). Multivariate parametric survival analysis (Weibull distribution) revealed pT-Stage, pN-Stage and high PD-L1 mRNA expression to be the only three parameters with a significant log worth concerning OS and DSS. Multivariate Cox-regression analysis including the same parameters as used in the Weibull distribution revealed that high mRNA-expression is an independent factor for a better outcome concerning overall survival (not for DSS).

PD-L1 mRNA values measured from CRNA in each lab (positive threshold: >36.5 40-ΔCt) and IHC analysis (1-step score) of P2 showed a substantial to almost perfect agreement (κ = 0.55–1.00; Figure 3A). IHC scoring and mRNA-expression (40-ΔCt) were matched in a heat map to investigate the divergence of IHC and mRNA positivity (Figure 3A). The heat map strongly suggests that there is a remarkable subset of tumors which exhibit substantial PD-L1 expression on mRNA level while no or no relevant protein expression could be observed by IHC (<1%/0%).

In order to validate this hypothesis, PD-L1 scoring (IC/TC) and PD-L1 gene expression analysis were performed in an independent cohort of 88 MIBC (Table 2; Figure 3B/3C). mRNA expression and IHC scoring shows good correlation which lies in the range found in the round robin test (*r =* 0.55–0.59, *p <* 0.0001; Figure 3B). The upper heat map visualizes that there is a large subgroup of mRNA positive tumors (*n =* 17; ≥36.5 40-ΔCt) which is IHC negative (<1% IC/TC/both). This effect is expanding if several diagnostic cut-offs are applied, e.g. the combined IC/TC cut-off of lower/higher than 10% which was utilized by Bellmunt *et al.* (*n =* 41; Supplementary Figure 1) [9]. Correlations and agreement of continuous IHC scoring and PD-L1 expression as well as correlations of PD-L1 mRNA expression ≥/< 36.5 40-ΔCt and various IHC cut-offs are depicted in Supplementary Table 5.

**Table 2:** Characteristics of the entire validation cohort (*n =* 88) as well as characteristics of the PD-L1 high/low and the PD-L1 IHC IC >/< 1% subgroups

Characteristic	Entire Cohort (*n =* 88)	PD-L1 ≥36.5 ∆Ct	PD-L1 < 36.5 ∆Ct	*p*-Value	PD-L1 IHC ≥ 1% (IC)	PD-L1 IHC < 1% (IC)	*p*-Value
**pT-Stage**				0.096			0.27
pT2	24 (27%)	13 (28%)	11 (26%)		8 (28%)	16 (27%)	
pT3	47 (53%)	28 (61%)	19 (45%)		18 (62%)	29 (49%)	
pT4	17 (20%)	5 (11%)	12 (29%)		3 (10%)	14 (24%)	
**pN-Stage**				0.014			0.019
pN0	60 (68%)	36 (78%)	24 (57%)		23 (79%)	37 (63%)	
pN1	9 (10%)	1 (2%)	8 (19%)		0 (0%)	9 (15%)	
pN2	19 (22%)	9 (20%)	10 (24%)		6 (21%)	13 (22%)	
**Grading**							
**WHO 1973**							
G2	4 (5%)	3 (7%)	1 (2%)	0.34	2 (7%)	2 (3%)	0.47
G3	84 (95%)	43 (93%)	41 (98%)		27 (93%)	57 (97%)	
	0 (0%)	0 (0%)	0 (0%)	1.0	0 (0%)	0 (0%)	1.00
**WHO 2016**	88 (100%)	46 (100%)	42 (100%)		29 (100%)	59 (100%)	
Low grade							
High grade							
**L1**	50 (57%)	22 (48%)	28 (66%)	0.074	13 (45%)	37 (62%)	0.11
**V1**	26 (30%)	11 (24%)	15 (36%)	0.23	7 (24%)	19 (32%)	0.43
**Pn1**	27 (30%)	17 (37%)	10 (24%)	0.18	12 (41%)	15 (25%)	0.13
**Carcinoma *in situ***	62 (70%)	32 (70%)	30 (71%)	0.85	17 (58%)	45 (76%)	0.092
**Median age** (Min./Max.)	70.2 (41.3–90.8)	70.1 (50.7–85.4)	70.7 (41.3–90.8)	0.45	70.05 (53.6–84.4)	70.28 (41.3–90.8)	0.64
**Adjuvant platinum-containing chemotherapy**	29 (33%)	14 (30%)	15 (36%)	0.60	8 (28%)	21 (36%)	0.45
**Median survival time** (Min./Max.)	26.6 (0.03–135.7)	39.7 (0.83–135.7)	14.53 (0.03–114.83)	0.0065	40.9 (0.08–135.7)	20.83 (0.03–129.4)	0.088
**Censors:**							
Alive	28 (32%)	18 (39%)	10 (24%)	0.12	12 (41%)	16 (27%)	0.18
Deceased	60 (68%)	28 (61%)	32 (76%)	0.12	17 (59%)	43 (73%)	0.18
Tumor specific death	37 (42%)	15 (33%)	22 (52%)	0.15	10 (34%)	27 (46%)	0.31

### Prognostic relevance of PD-L1 IHC and gene expression

**Figure 4:**
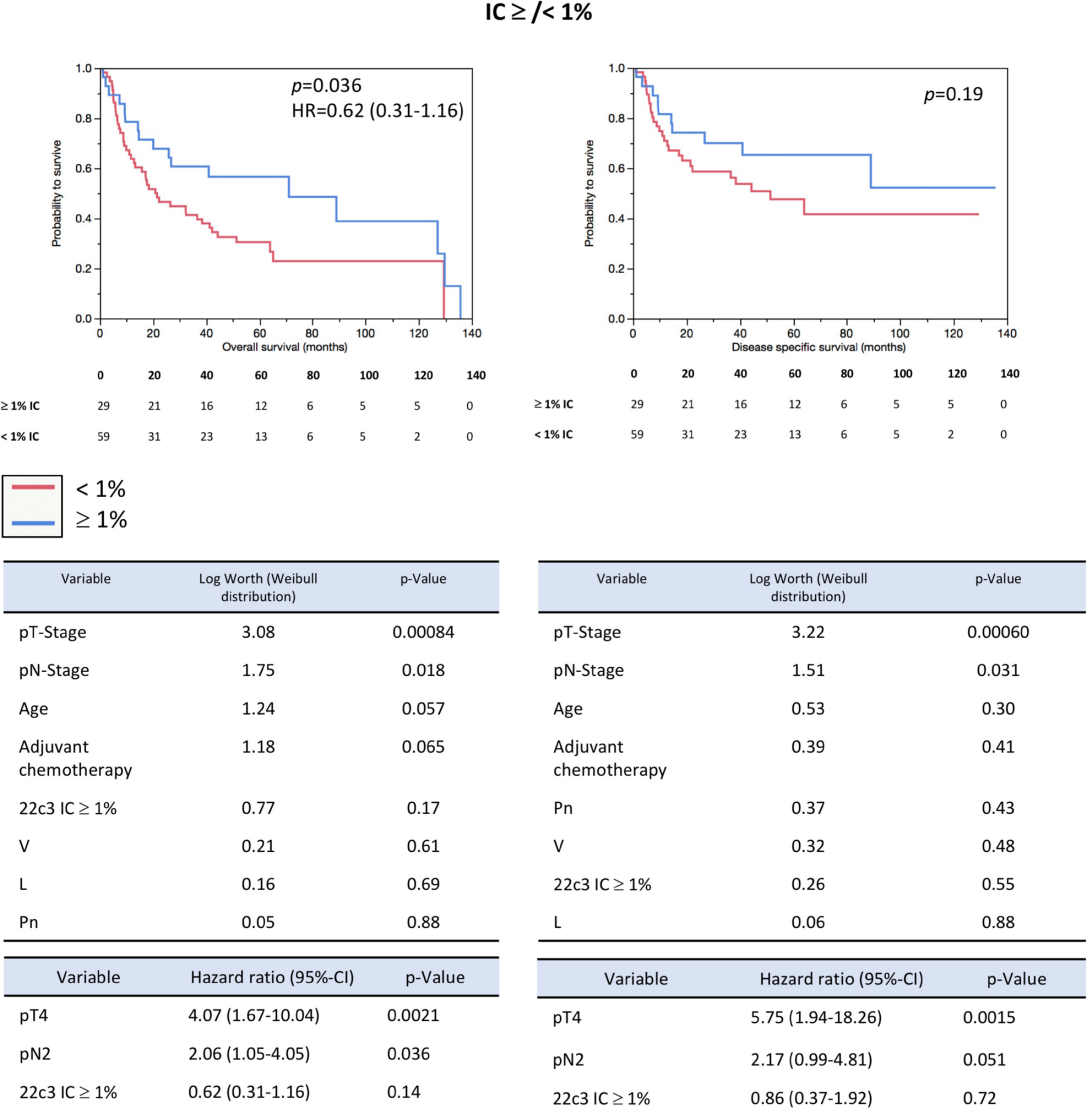
Prognosis prediction (PD-L1 IHC) Kaplan–Meier-analysis revealed that PD-L1 IHC IC 1% cut-off prognostic concerning OS, but not DSS (OS: log rank *p =* 0.036; HR *=* 0.62 [95%-CI 0.31–1.16]; DSS: log rank *p =* 0.19). Multivariate parametric survival analysis (Weibull distribution) revealed pT-Stage and pN-Stage to be the only two parameters with a significant log worth concerning OS and DSS. Multivariate Cox-regression analysis including the same parameters as used in the Weibull distribution revealed that high PD-L1 expression on IC of ≥1% is not an independent risk factor.

Due to the significantly higher rate of PD-L1 positivity on gene expression level, comparative survival analysis was performed (Table 2; Figure 3C, Supplementary Figure 1). Patients with mRNA expression ≥36.5 40-ΔCt had a significantly better OS (5 year-OS: 50% vs. 26%, *p =* 0.0042) and DSS (5 year DSS: 65% vs. 40%, *p =* 0.012). Concerning IHC scoring, only the IC ≥/< 1% cut-off revealed a significant better OS (5-year OS: 58% vs. 31%, *p =* 0.036, HR *=* 0.62; Figure 4). In multivariate parametric survival distributions (Weibull) high mRNA expression correlated significantly with better OS and DSS (Figure 4C) while IHC cut-offs revealed no significance neither for OS nor for DSS (Figure 4; Supplementary Table 4). In a multivariate Cox-regression model high PD-L1 expression was an independent positive prognostic factor concerning OS (HR *=* 0.48, *p =* 0.019; Figure 3C) whereas it was not concerning DSS (HR *=* 0.49, *p =* 0.066). The 1% IHC IC cut-off revealed no significance in multivariate analysis for OS (HR *=* 0.62, *p =* 0.14) nor for DSS (HR *=* 0.86, *p =* 0.72; Figure 4).

## DISCUSSION

Current studies revealed divergent results with regard to therapy responsiveness depending on IHC PD-L1 expression status. In particular, high IHC PD-L1 expression on IC/TC was partially associated with better objective response rates, but not with survival [9–12, 16]. Inherent of the detection method, semi-quantitative IHC is limited in its sensitivity and dynamic range compared to fully quantitative molecular assays such as RT-qPCR. Similar predictive IHC assays– e.g. Her2neu or Ki67 scoring– are affected by a large inter-observer variability that has important clinical implications [14]. Additional to inter-observer variability, PD-L1 scoring is also affected by biological diversity of commonly used assays leading to significantly different staining patterns [17–19]. Therefore, it seems to be necessary to establish methods for an objective and precise evaluation of PD-L1 status.

For breast cancer the value of determining Her2/neu and hormone receptor status with RT-qPCR has been demonstrated [20]. Risk assessment of hematological diseases such as CML has long been carried out on the basis of RT-qPCR, which could even be standardized internationally [21, 22].

**Figure 5:**
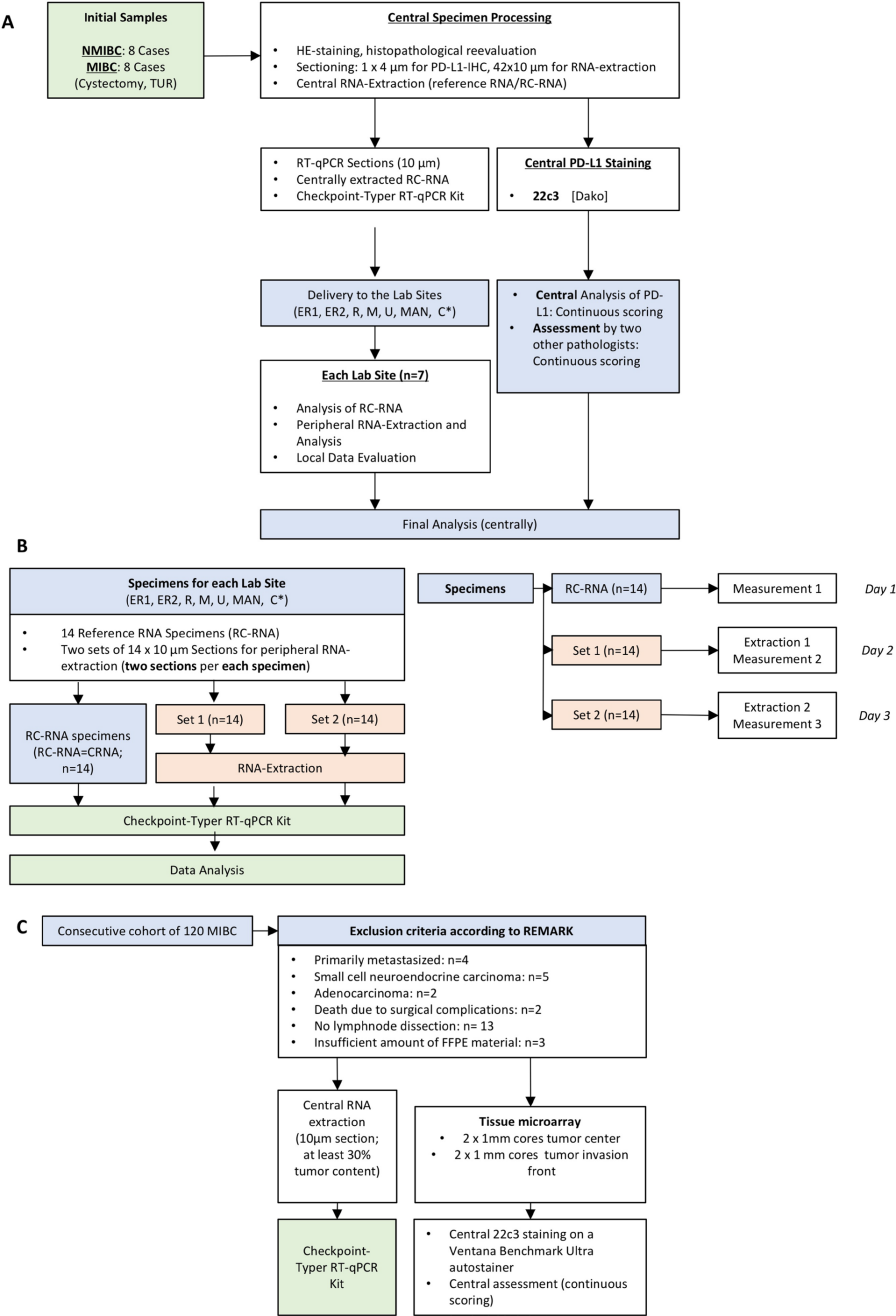
Sample selection, round robin test design and selection of sensitivity validating cohort **(A)** Design of the Round Robin Test. ^*^ER1: Department of Pathology, FAU Erlangen, ER2: Department of Urology, FAU Erlangen, R: Department of Urology, University of Regensburg, M: Department of Pathology, TU Munich, U: Department of Urology, University of Ulm, MAN: Department of Urology Mannheim, RKU Heidelberg, C: Institute of Molecular Pathology, Cologne. **(B)** Simplified work flow in the participating laboratory Sites. RC-RNA = Reference-Control-RNA (=centrally extracted RNA/CRNA); Set 1 & 2 = sections for peripheral RNA extraction on day 1 and 2. Specified work flow in the peripheral lab sites is depicted on the right: The protocol was conceived for three days. On day 1 the CRNA was measured whereas on day 2 and day 3 RNA was extracted in the lab sites and then measured. (RC-RNA = reference control-RNA; RT-qPC*R =* reverse transcriptase quantitative polymerase chain reaction). **(C)** Selection criteria (according to REMARK) of the consecutive MIBC validation cohort (*n =* 88). RNA extraction and tissue microarray (TMA) preparation were performed using the same FFPE block with tumor content of at least 30%. PD-L1 staining was carried out on Ventana Benchmark Ultra autostainer.

Here we tested the feasibility of RT-qPCR-based PD-L1 mRNA measurement in a multicentric round robin test (Figure 5A). Importantly, two critical concerns were addressed with satisfying results: (I) due to high standardization neither a significant influence of RNA extraction modality nor of technical variations of different thermocyclers appeared (Figure 2A); (II) inter-center reproducibility was high to extremely high (CRNA: *ρ* = 0.68–0.98, *p* ≤ 0.0076; LRNA: *ρ* = 0.81–0.98, *p* ≤ 0.014; Supplementary Table 2), which resulted in very low mean differences not exceeding –1.0 or 1.0 resembling very strong agreement (Figure 2B; Supplementary Table 3). Additionally, we investigated the “inter-specimen” divergence of PD-L1 expression (IHC/gene expression) in four matched pairs of TUR-B and CX. In two matched pairs, protein and gene expression was higher in TUR-B specimens while it was the opposite in the two other pairs (Figure 2A; Table 1). Although this is a very limited size of matched pairs, this might be an important observation towards tissue based therapy stratification. For example, expression analysis in specimens with low amount of tissue (TUR-B) with “artificially” high infiltration of lymphocytes caused by a sampling bias might lead to a wrong PD-L1 positivity status. This could happen in the opposite direction and lead to wrong PD-L1 negativity. Accumulation of such cases might distort study results in relevant manner. Therefore, further studies are needed to investigate this potential bias especially in cohorts of patients receiving checkpoint inhibitor therapies.

To investigate the relationship between RT-qPCR and IHC, we compared RT-qPCR data with IHC scoring of three experienced pathologists who had not previously received systematic training. IC and combined IC/TC scoring revealed moderate to substantial agreement (Figure 2C), which is presumably caused by low TC positivity and consecutive easement in assessing IC as positive or negative. TC scoring revealed slight to fair agreement. Agreement for IC scoring was clearly higher than previously reported although the observer received no systematically training (Figure 1B) [23]. RT-qPCR intra class correlation was very strong (Figure 2A, Supplementary Table 2). While inter-method agreement for TC/IC scoring (1% cut-off) ranged from fair to almost perfect in the round robin setting (Figure 1B), inter-method agreement decreased to substantial and slight depending on applied cut-offs in the validation cohort (Supplementary Table 5). This is owed to a gap (mRNA vs. IHC positivity) which expands significantly if different cut-offs for IHC are applied (Figure 3B; Supplementary Table 5). Taken together, inter-method agreement/correlation seems to range in acceptable dimensions for certain cut-offs.

Nevertheless, reproducibly in all centers, RT-qPCR assessment resulted in more PD-L1 positive samples than IHC scoring which was validated in 88 MIBC specimens (Figure 3A–3B). This gap is expanding by applying higher cut-offs than ≥/< 1% (Figure 4B). Regarding immunohistochemistry, this effect is also observable in a recently published clinical trial of Bellmunt *et al*: While 27% (142/526; our study: 33% [29/88]) of all Pembrolizumab treated patients were positive for the combined 1% IC/TC cut-off, the amount of positive patients decreased to 20% for the combined 10% IC/TC cut-off (104/526; our study: 6% [5/88]) [9]. The remarkable difference of positive cases for the combined 10% cut-off is probably caused by our limited cohort size of 88 cases and a potential lower amount of highly positive tumors. This observation is of particular interest due to a significantly increased OS and DSS in the mRNA positive subgroup (Table 2; Figure 3C) while IHC showed only significant prognosis prediction for 1% IC cut-off concerning OS (Figure 4). Concerning OS mRNA expression was also an independent positive prognostic factor in a multivariate Cox regression model (HR *=* 0.48, *p =* 0.019; Figure 3C). The found prognostic positive influence of PD-L1 expression is congruent with previous reports [24, 25]. However, previous studies have shown that high expression of PD-L1 is also linked to aggressive tumor behavior and higher tumor stage in UBC and other cancer entities [26–28]. Possibly, high expression of PD-L1 might be an indicator for a strong preexisting anti-tumor immunity which could eliminate residual tumor cells after radical cystectomy more efficient than a lowly activated immune system. This could explain why patients with high PD-L1 expression have a favorable OS and DSS.

Although the gap between mRNA and IHC positivity appears to be a true effect with prognostic significance, the magnitude of the effect must be critically evaluated. Assuming that the cut-off for mRNA positivity would have been set for technical reasons, the effect size would have been significantly smaller. To prove the size and relevance of the gap effect further investigations with internal standards are needed. Despite technical issues this gap between mRNA and protein expression could also be a hint that the translation of PD-L1 mRNA might be inhibited in these tumors. As demonstrated in previous studies, gene and protein expression of PD-L1 is mainly regulated by interferons- mainly by interferon gamma- and stabilized by TNF-alpha [29, 30]. Other factors such as the ubiquitin-ligase regulating protein CSN5 are able to further stabilize the PD-L1 protein [31]. Therefore, currently undiscovered processes affecting the stabilization of PD-L1 but not affecting the gene expression might lead to IHC PD-L1 negative tumors which are truly positive. Additionally, similar mechanisms as the previous described disruption of the 3′-UTR in the PD-L1 gene might lead to affections of diagnostic antibody body binding sites leading to a false negativity with upregulation of PD-L1 on both, gene and protein level [32]. In the light of differing results across different clinical trials concerning therapy responsiveness toward checkpoint inhibitors, upcoming studies are need investigating the role of PD-L1 regulation in patients receiving checkpoint inhibitors.

In conclusion, several aspects could be demonstrated:

(I) the reproducibility of both methods is acceptable, although it is much better for mRNA expression regardless of utilized thermocyclers or RNA extraction modality.

(II) The mRNA-based PD-L1 assessment resulted in a significantly higher rate of positive cases leading to superior prognosis prediction in a large cohort of MIBC treated with radical cystectomy. Against the background of these results, the mRNA-based PD-L1 determination therefore appears to be a possible, highly reproducible and objective method. As the observed responsiveness of “PD-L1 negative” patients determined by IHC might be related to the lack of detecting positive cases due to several reasons, mRNA expression detection might possibly identify those patients [10–12]. However, this point has to be addressed in upcoming studies comparing gene expression and IHC scoring towards responsiveness of checkpoint inhibitor therapies.

Results of the present study are limited by limited sample size, sampling of the FFPE material for RNA extraction which might affect gene expression results in comparison to fresh frozen tissue. Furthermore, comparisons of protein expression and gene expression are limited by the fact that gene expression levels can differ greatly from protein expression of the distinct gene and vice versa. Results of prognosis prediction are limited due to the fact that the two diagnostic tests were compared in a cohort of curative treated patients, and not in a cohort of patients receiving checkpoint inhibitors why further head to head comparisons of their performance as predictive biomarker are an important further step.

## MATERIALS AND METHODS

### Patient population and specimen collection

For the round robin test formalin fixed paraffin embedded (FFPE) tumor tissue samples from 16 patients were obtained: 8 cases of muscle-invasive bladder cancer (MIBC; pT2-4, radical cystectomy/RC) and the corresponding transurethral resections (TUR; *n =* 16), 8 cases NMIBCs (pT1, TUR; *n =* 8). The final cohort with adequate tissue quality consisted of 4 NMIBC (pT1; G2/high grade [*n =* 2]; G3/high grade [*n =* 2]) and 10 MIBC (all G3/high grade; TUR-specimens *n =* 6; RC-specimens *n =* 4).

A consecutive cohort of 88 patients from a single center with MIBC (2000-2011) treated with RC and lymphonodal dissection was investigated to compare sensitivity and prognostic relevance of both assays (mRNA and IHC). Exclusion criteria, cohort and subgroup characteristics are depicted in Table 2 and Figure 5C.

All specimens were reevaluated by an experienced uropathologist (AH) according to the latest TNM (2017) and WHO classification (2016). All patients gave informed consent. All experiments were performed in accordance with the Helsinki declaration of 1975.

### Sample preparation and tissue micro array construction

Round robin test specimens: 1 × 4 µm section per specimen for PD-L1 IHC (22c3, DAKO) and 14 × 10 µm sections per specimen for LRNA-extraction (Figure 5A). Centrally extracted RNA was extracted out of 10 × 10 µm sections to obtain a sufficient amount of RNA for distribution to all participating lab sites. The tumor content was at least 30% with a minimal tumor size of 5 × 5 mm.

Validation cohort: A representative FFPE block with at least 30% tumor content (minimal tumor size 5 × 5 mm), a well delimited invasion border, and without necrosis areas or granulomatous inflammation was selected. For PD-L1 IHC scoring a tissue microarray (TMA) was prepared: HE slides were scanned (Panoramic P250, 3DHistech, Hungary) and annotated using a TMA annotation tool (Panoramic viewer v15.1.). Four cores (2× tumor center, 2× invasion front; diameter 1mm) were taken utilizing an automated tissue microarrayer (TMA Grandmaster, 3DHistech, Hungary) as described previously [33].

### RNA isolation from formalin-fixed paraffin-embedded tissue and Checkpoint Typer Kit

RNA was extracted based on a magnetic bead technology using a single 10 µm FFPE section (STRATIFYER, Molecular Pathology GmbH, Cologne, Germany). Sections were solubilized, paraffin was melted and tissue was lyzed with Proteinase K. Lysates were admixed with germanium-coated magnetic particles in buffer-controlled conditions. Purification was carried out by means of 3 consecutive washing cycles involving magnetization, centrifugation, washing and removal of the supernatant. Expression levels of PD-L1 and CALM2 were assessed in triplicates by RT-qPCR (Lab 1, 2 and 7: LightCycler 480, Roche; Lab3 and 5 Step-One-Plus, Applied Biosystems; Lab4 Biorad CFX, Biorad; Lab 6: VIIA7, Applied Biosystems) using the Checkpoint Typer Kit (STRATIFYER) [34].

To obtain sufficient data, all specimens were tested for the constitutively expressed gene Calmodulin 2 gene (CALM2) which is known as a stable reference/housekeeper gene [34]. Specimens with Ct value of CALM2 values of higher than 28 were excluded.

### Validation of the checkpoint typer kit

For technical validation of respective cut off values a PD-L1^+/-^ cell line derived TMA created by Horizon Discovery (Cambridge, United Kingdom) was used. Separate measurements of 1 or 3 TMA cuts of both cell lines were analyzed by the Checkpoint Typer Kit as described above (Figure 1A). To verify the results, the positive/negative Horizon TMAs were stained immunohistochemically by the 22c3 assay which has been utilized in this study (DAKO; Figure 1A).

### PD-L1 immunohistochemical assay and analysis

All specimens (round robin test, validation cohort) were stained with PD-L1 assay 22c3 (Dako, Carpinteria, CA, USA) according to manufacturer’s instructions on a Ventana Benchmark Ultra automated slide stainer (Ventana, Tucson, Arizona, USA; Figure 5A). The round robin test specimens were scored discretely (0–100%; 1% steps from 0–10%; 5% steps from 10–100%) for IC and TC expression by three experienced pathologists (AH, WW, SE). For inter-observer variability as well as sensitivity and prognosis prediction analysis several commonly used scores were applied (Figure 1B) [9, 10, 12, 16]. TMA stainings (validation cohort) were analyzed by AH and ME.

### Round robin test design

In detail, the round robin test design with 7 participating centers is depicted in Figure 5B. Specimens were sectioned centrally, reference control RNA (RC-RNA = CRNA) was extracted and other section sets were sent to the lab sites. All labs were instructed and trained for one day by an experienced laboratory technician. The section sets 1 and 2 were processed in the labs on different days. Data were analyzed centrally.

### Statistical analysis

Statistical analysis was performed using JMP13.1 (SAS, Cary, North Carolina, USA) and GraphPad Prism 5 (San Diego, California, USA). Correlations between variables were investigated using the Spearman’s rank correlation coefficient (Rho) and scatter plots, Mann-Whitney *U* test or Fishers exact test, whichever was appropriate. Inter-observer and inter-method comparison was investigated using the Light’s Kappa method (Figure 1B) [35]. To compare reproducibility and systematic differences Bland-Altman-analysis was performed. This method shows the difference between measurements (e.g. various labs, CRNA vs. LRNA extraction) against their mean to indicate whether there is a systematic difference between the measurements. The 95% limits of agreement can provide an interval in which most of the individual differences between measurements could be expected to lie [36, 37]. For comparing the sensitivity of IHC and RT-qPCR hierarchical cluster analysis were performed using Euclidean distance and the average linkage algorithm.

For survival analysis Kaplan–Meier analysis were performed and significance was tested by the log-rank test. Parametric survival analysis was performed using the Weibull-distribution in a multivariate model. Hazard ratios were calculated by in multivariate model by Cox-regression. *p*-Values of < 0.05 were considered to be significant. All tests were two-sided.

## SUPPLEMENTARY MATERIALS FIGURE AND TABLES







## Abbreviations

PD-L1: programmed death ligand 1
BC: bladder cancer
UBC: urothelial bladder cancer
CRNA: centrally extracted RNA
LRNA: locally extracted RNA
RC: radical cystectomy
RT-qPCR: reverse transcriptase quantitative polymerase chain reaction
IC: immune cells
TC: tumor cells
NSCLC: non-small cell lung carcinoma
MIBC: muscle invasive bladder cancer
NMIBC: non muscle invasive bladder cancer
CALM2: calmodulin 2
TUR: transurethral resection
FFPE: formalin fixed paraffin embedded
Ct: cycle threshold
TMA: tissue microarray
