# Supplementary material for: A multicenter round robin test of PD-L1 expression assessment in urothelial bladder cancer by immunohistochemistry and RT-qPCR with emphasis on prognosis prediction after radical cystectomy
Source: Oncotarget. 2018 Feb 19;9(19):15001–14. doi: 10.18632/oncotarget.24531 (PMC5871092; doi:10.18632/oncotarget.24531)
Supplement: Supplementary file 1 [file oncotarget-09-15001-s001.pdf]

A multicenter round robin test of PD-L1 expression assessment in urothelial bladder cancer by immunohistochemistry and RT-qPCR with emphasis on prognosis prediction after radical cystectomy

SUPPLEMENTARY MATERIALS

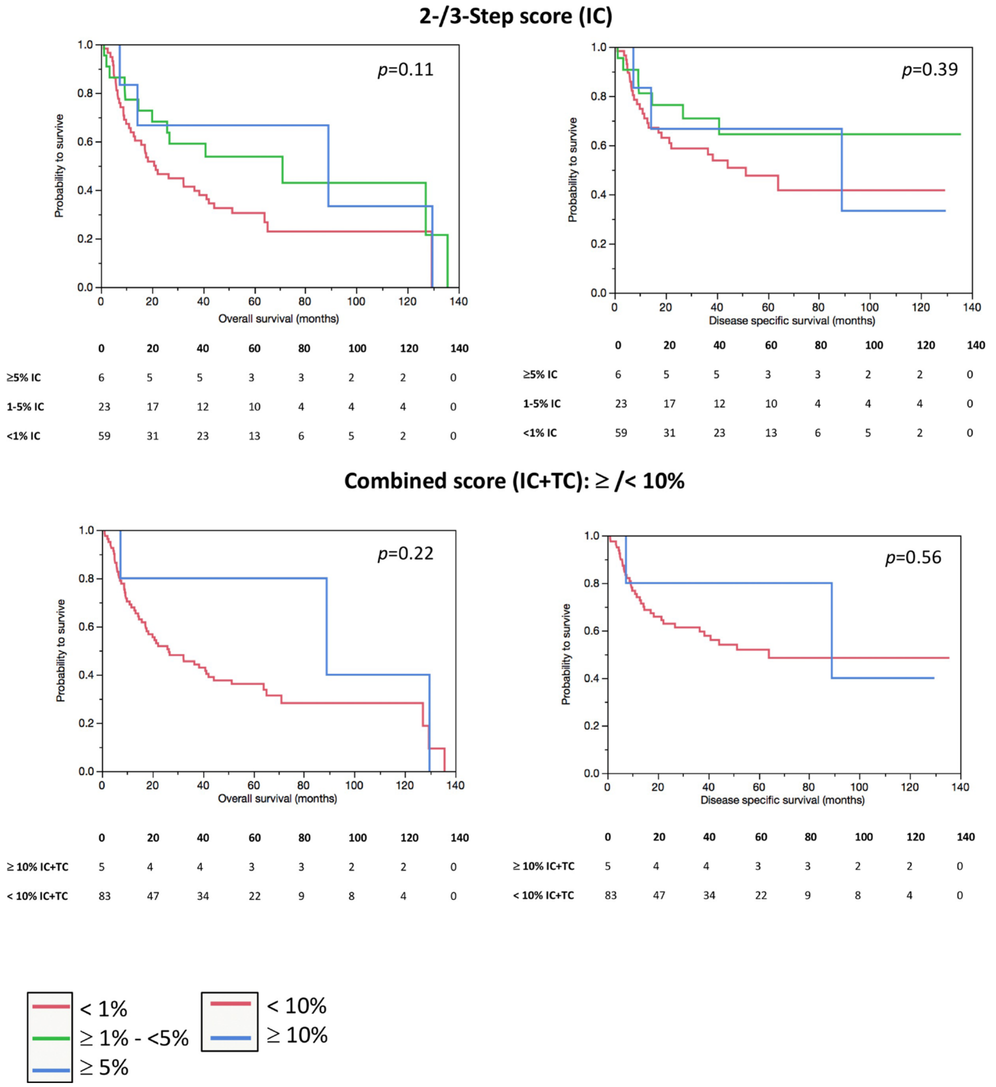

Supplementary Figure 1: Kaplan-Meier-analysis of differential immunohistochemical cut-offs. Kaplan-Meier-analysis for different IHC cut-offs which were utilized in clinical trials 9,12. For 2-/3-Step (IC scoring) and combined IC + TC cut-off there was no significant better prognosis in the high expression groups. No cut-off reached significance in the multivariate Weibull distribution model why no multivariate Cox regression analysis was performed (Supplementary Table 4).

Supplementary Table 1: Immunohistochemical single case evaluation of each participating pathologist. See Supplementary_ Table_1

Supplementary Table 2: Inter-center correlation of PD-L1 mRNA expression measurements

A

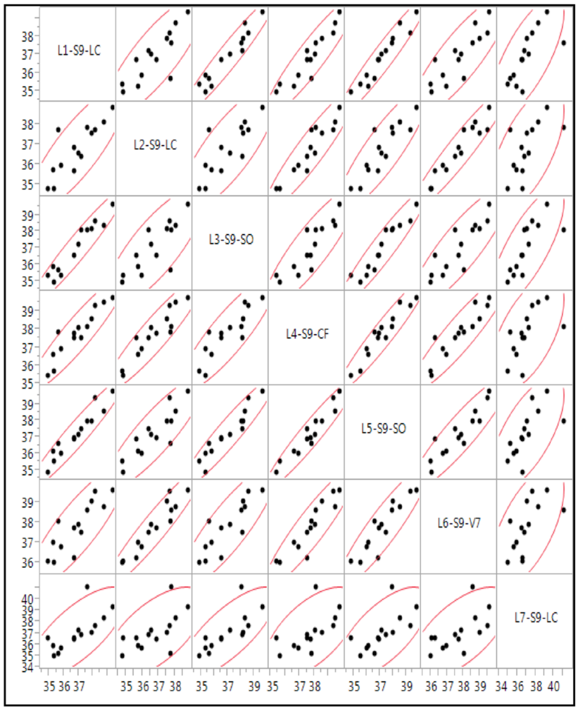

Spearman's ρ: Interlab correlation - Centrally Extracted RNA			
**Lab A**	**Lab B**	**ρ**	**> |ρ|**
L5	L3	0.98	<0.0001
L5	L1	0.97	<0.0001
L6	L4	0.95	<0.0001
L3	L1	0.94	<0.0001
L5	L4	0.93	<0.0001
L6	L2	0.93	<0.0001
L4	L1	0.92	<0.0001
L6	L5	0.91	<0.0001
L4	L2	0.91	<0.0001
L4	L3	0.88	<0.0001
L6	L3	0.88	<0.0001
L6	L1	0.86	<0.0001
L7	L3	0.86	<0.0001
L7	L5	0.85	0.0001
L7	L1	0.84	0.0002
L5	L2	0.82	0.0003
L2	L1	0.80	0.0006
L7	L4	0.78	0.0011
L3	L2	0.77	0.0014
L7	L6	0.72	0.0035
L7	L2	0.68	0.0076

B

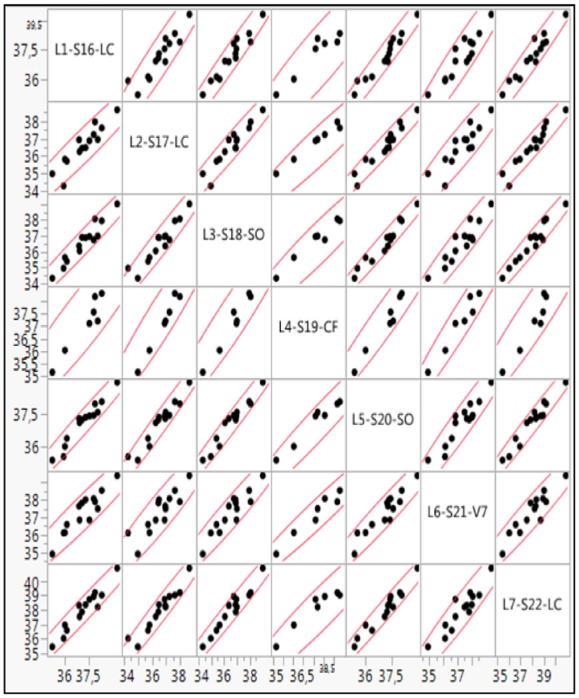

Spearman's ρ: Interlab correlation - Locally Extracted RNA			
**Lab A**	**Lab B**	**ρ**	**> |ρ|**
L2	L1	0.93	<0.0001
L3	L1	0.95	<0.0001
L3	L2	0.91	<0.0001
L4	L1	0.90	0.0068
L4	L2	0.96	0.0005
L4	L3	0.86	0.014
L5	L1	0.98	<0.0001
L5	L2	0.97	<0.0001
L5	L3	0.95	<0.0001
L5	L4	0.97	0.0005
L6	L1	0.86	<0.0001
L6	L2	0.88	<0.0001
L6	L3	0.81	0.0004
L6	L4	0.97	0.0005
L6	L5	0.88	<0.0001
L7	L1	0.90	<0.0001
L7	L2	0.96	<0.0001
L7	L3	0.90	<0.0001
L7	L4	0.93	0.0025
L7	L5	0.95	<0.0001
L7	L6	0.90	<0.0001

Measuring centrally **(A)** and locally **(B)** extracted RNA the most labs showed high intraclass correlation in pair comparisons (**A**: $\rho = 0.68–0.98$, $p \leq 0.0076$; **B**: $\rho = 0.81–0.98$, $p \leq 0.014$).

Supplementary Table 3: Explicit graphical and numerical illustration of Bland-Altman-analysis of inter-center agreement

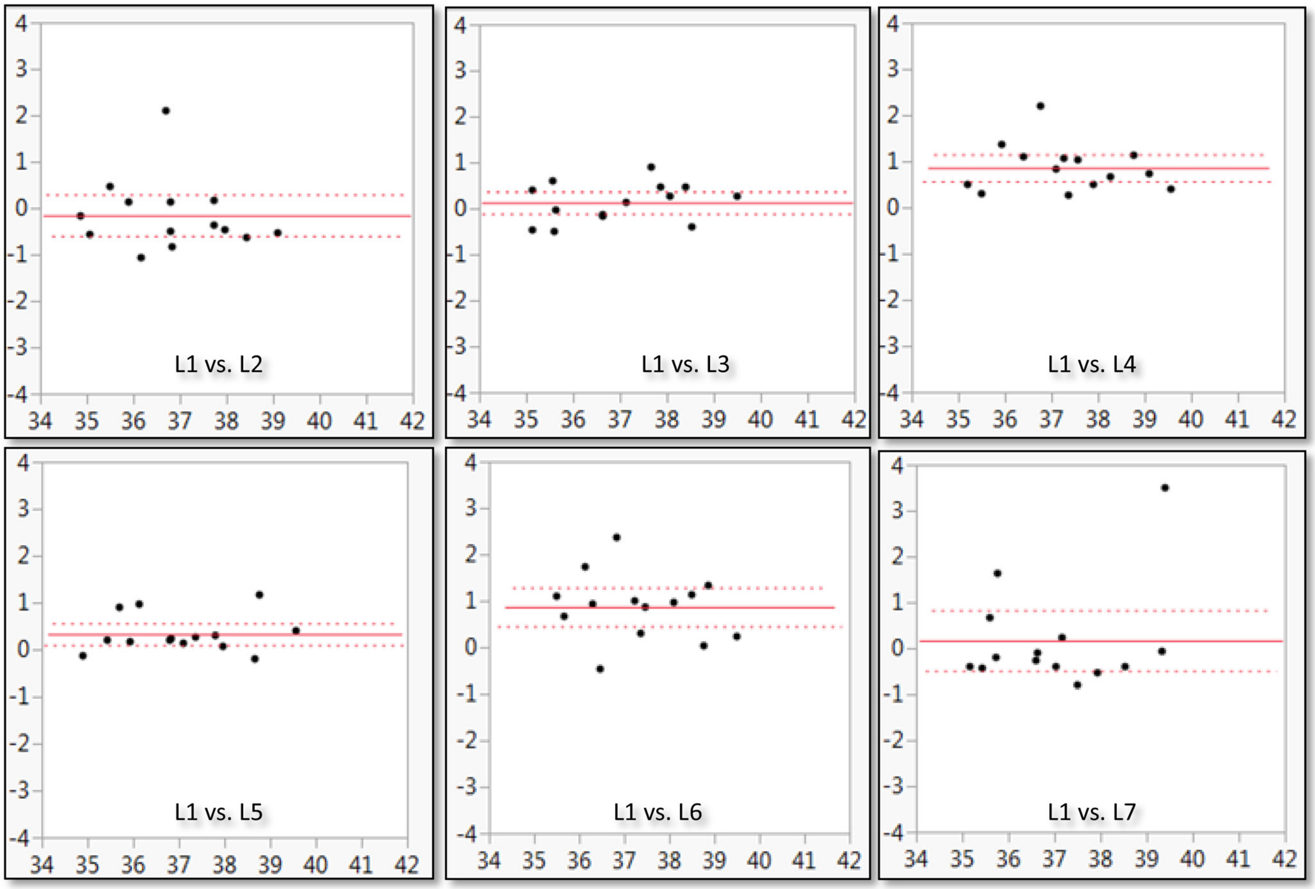

	L1 vs. L2	L1 vs. L3	L1 vs. L4	L1 vs. L5	L1 vs. L6	L1 vs. L7
n	14	14	14	14	14	14
Mean	0.15	-0.13	-0.86	-0.33	-0.87	-0.17
Median	0.42	-0.21	-0.78	-0.21	-0.94	0.23
Maximum	1.06	0.50	-0.28	0.20	0.46	0.81
Minimum	-2.09	-0.89	-2.19	-1.17	-2.38	-3.51
Std. Deviation	0.77	0.42	0.51	0.40	0.72	1.14
Std. Error	0.21	0.11	0.14	0.11	0.19	0.30
Lower 95% CI of mean	-0.30	-0.37	-1.16	-0.56	-1.28	-0.83
Upper 95% CI of mean	0.60	0.12	-0.57	-0.097	-0.46	0.49

Supplementary Table 4: Multivariate parametric Weibull-distribution analysis of mRNA expression and different immunohistochemical cut-offs. See Supplementary_Table_4

Supplementary Table 5: Intra-class correlation and inter-method concordance in the sensitivity validation cohort

Cut-off	Light's κ (IC)	Light's κ (TC)	Light's κ (IC+TC)
≥/< 1%	0.62	0.58	0.62
≥/< 5%	0.13	0.10	0.32
≥/< 10%	0.06	0.06	0.10
2/3-Step	0.52	0.49	0.39

B

mRNA	IHC	Spearman *r*	*p*-Value
PD-L1 mRNA	IC ≥/< 1%	0.67	*<0.0001*
PD-L1 mRNA	IC ≥/< 5%	0.26	*0.015*
PD-L1 mRNA	IC ≥/< 10%	0.18	*0.094*
PD-L1 mRNA	2/3-Step	0.66	*<0.0001*
PD-L1 mRNA	TC≥/< 1%	0.64	*<0.0001*
PD-L1 mRNA	TC ≥/< 5%	0.23	*0.028*
PD-L1 mRNA	TC ≥/< 10%	0.18	*0.094*
PD-L1 mRNA	2/3-Step	0.63	*<0.0001*
PD-L1 mRNA	IC+TC ≥/< 1%	0.67	*<0.0001*
PD-L1 mRNA	IC+TC ≥/< 5%	0.43	*<0.0001*
PD-L1 mRNA	IC+TC ≥/< 10%	0.23	*0.028*
PD-L1 mRNA	2/3-Step	0.66	*<0.0001*

mRNA	IHC	Spearman r	*p*-Value
Continuous PD-L1 mRNA	Continuous IC scoring	0.59	*<0.0001*
Continuous PD-L1 mRNA	Continuous TC scoring	0.59	*<0.0001*
Continuous PD-L1 mRNA	Continuous IC + TC scoring	0.55	*<0.0001*

Agreement and intra-class correlation analysis in the independent validation cohort (*n* = 88). (**A**) Light's Kappa Analysis for PD-L1 mRNA ≥/< 36.5 40-ΔCT versus various IHC cut-offs. Agreement is moderate to substantial for IC/TC ≥/< 1% cut-off. For other cut-offs agreement ranges from slight to fair. (**B**) Spearman correlations PD-L1 mRNA ≥/< 36.5 40-ΔCT versus various IHC cut-offs. The intra-class correlations are especially high for ≥/< 1% cut-offs and for the 2/3-Step cut-off utilized in the IMvigor trials 1,2. Continuous PD-L1 mRNA expression and continuous PD-L1 scoring also show a high inter-method correlation
